# Invasive mechanical ventilation complications in COVID-19 patients

**DOI:** 10.1186/s43055-021-00609-8

**Published:** 2021-09-20

**Authors:** Ghada Sobhy Ibrahim, Buthaina M. Alkandari, Islam Ahmed Abo Shady, Vikash K. Gupta, Mohsen Ahmed Abdelmohsen

**Affiliations:** 1 Department of Radiodiagnosis, Faculty of Medicine, Tanta University, Algeish St, Tanta, 31527 Egypt; 2 FFR-RCSI, Dublin, Ireland; 3 Jaber Al Ahmad Hospital, Ministry of Health, Khalid Ben Abdulaziz Street, South Surra, Kuwait City, Kuwait; 4 Department of Radiodiagnosis, Faculty of Medicine, Mansora University, Mansora, Egypt; 5 University of Rajasthan, Rajasthan, India; 6 Present Address: Department of Radiodiagnosis and Intervention, Faculty of Medicine, University of Alexandria, 10 Shamplion Street, Elazareeta, Alexandria, Egypt

**Keywords:** Barotraumas, COVID-19 pneumonia, Invasive mechanical ventilation

## Abstract

**Background:**

Since late 2019, COVID-19 infection has quickly spread substantially in all countries, forcing the appropriation of noteworthy lockdown and social separating measures. It has been considered as a pandemic by the World Health Organization. Positive pressure ventilation is a non-physiological and invasive intervention that can be lifesaving in COVID-19 patients. Similar to any other interventions, it can cause its own danger and complications as it can prompt ventilator-induced lung injury and barotrauma. The aim of the work was to identify the incidence of invasive mechanical ventilation complication in COVID-19 pneumonias, and to describe patient characteristics and patterns of barotrauma in COVID-19 patients.

**Results:**

This retrospective study included 103 patients with COVID-19 pneumonia, 76 males and 27 females are on invasive mechanical ventilation. Their mean age was 56.6, ranged from 21 to 85 years old. Barotraumas event type in the studied patients, (NB: one or multiple barotrauma events occurring on the same day were considered as single event (95/103 patients-92.23%), while separate multiple events (8/103 patients-7.77%) were recorded when occurring separated by at least 24 h). Single barotrauma events were subdivided into*:* one event (67/95 patients—70.53%), & multiple events (28/95 patients—29.47%). The mean interval between invasive mechanical ventilation and developing barotraumas was 3–7 days included 41 patients (39.98%). We revealed a strong prevalence of COVID-19 IMV complication with worsening prognosis and subsequent higher death rates in elderly smoker or obese males, as well as those suffering from ARDS. Past medical history (hypertension, DM, chronic renal or cardiac disease) or surgical history of CABG was more liable for these types of complications.

**Conclusion:**

Patients with COVID-19 pneumonia were more liable to the higher incidence of barotraumas with presence of predisposition and high risk factors. In general, an outstanding bad prognostic outcome and a significantly high mortality rate prevailed in COVID-19 patients associated with mechanically ventilated patients.

## Background

Coronavirus disease 2019 (COVID-19) is a newly identified infectious disease that caused by severe acute respiratory syndrome corona-virus 2 (SARS-CoV-2) which is a latterly rising zoonotic agent. Since late 2019, COVID-19 infection has quickly spread substantially in all countries, forcing the appropriation of noteworthy lockdown and social separating measures. It has been considered as a pandemic by the World Health Organization (WHO) [1, 2].

As data collected from Chinese Center for Disease Control and Prevention, the range of infirmity went from gentle malady (no or mellow pneumonia) in 81% with a standard convalescent time of around 14 days, through extreme sickness (dyspnea, hypoxia or > 50% lung association on imaging inside 24–48 h) in 14% with a recuperation time of around 2 month to critical ailment (ARDS, sepsis or septic shock) in 5%; this was seen in information of 44,500 affirmed cases of COVID-19 [3].

The most common complications in COVID-19 infections are bilateral pneumonia which may progressed to ARDS, sepsis and septic shock, acute kidney injury and others such as acute cardiac injury (arrhythmias, myocardial infarction,heart failure), coagulopathy, hyponatremia and acidosis. Complications are more in serious sickness versus non-extreme illness [4].

Positive pressure ventilation is a non-physiological and invasive intervention that can be lifesaving in COVID-19 patients. Similar to any other interventions, it can cause its own danger and complications as it can prompt ventilator-induced lung injury and barotrauma [5].

Due to pressure difference in enclosed cavities within the body, barotrauma can damage body tissue. Sinuses (sinus barotrauma), middle ear (otic barotrauma) and the lungs (pulmonary barotrauma) are the most commonly affected organs by barotrauma [6].

Pulmonary barotrauma in mechanical ventilation alludes to alveolar burst because of increasing trans-alveolar pressure (the alveolar pressure minus the pressure in the adjacent interstitial spaces), that leads to air spills into extra-alveolar spaces that causes pneumothorax, subcutaneous emphysema, pneumomediastinum and pneumoperitoneum. It is related to increase in morbidity and mortality [7].

The incidence of barotrauma changes with the underlying indication for mechanical ventilation, and several past studies found patients with underlying lung disease (Pneumonia, Chronic interstitial lung disease COPD, ARDs) more susceptible to barotrauma [7].

Pulmonary barotrauma related to mechanical ventilation is the condition that commonly occurs in ICU patients. It leads to increase morbidity and mortality. The ICU medical attendants caring for patients on mechanical ventilators must be fully aware of barotrauma and how to react. The condition can bring about a life-threatening tension pneumothorax and lead to death within minutes. Medical attendants need to survey the patient's ventilatory parameters, frequently palpate the chest for crepitus, then check the chest X-rays [8].

### Aim of the work

To identify the incidence of invasive mechanical ventilation complication in COVID-19 pneumonias, impact on the clinical outcomes and to describe patient characteristics and patterns of barotrauma in COVID-19 patients.

## Methods

Retrospective evaluation of Chest X-ray (CXRs) and Multi-detector Computed Tomography (MDCT) of the chest to detect invasive mechanical ventilation complications of all confirmed COVID-19 patients with positive RT-PCR for SARS-Cov-2 nucleic acid on nasopharyngeal throat swabs in a period extending from March 1, 2020 to November 1 2020 will be done. All CXRs of these patients were taken by portable digital radiography X-ray machines (GE OPTIMA XR 220, SIEMENS Mobile Elara Max & PHILIPS Mobile Diagnost wDR) with standard department exposure protocols of Medical Imaging Department-Diagnostic Radiology of our institution.

### Inclusion criteria

Confirmed patients with COVID-19 infection by positive PCR test for the causative agent (SARS-COV-2 virus) who presented with invasive mechanical ventilations complications.

### Exclusion criteria

Non mechanically ventilated Confirmed COVID-19 patients

Non hospitalized confirmed COVID-19 patients

The recorded demographic and clinical features included: age, gender, time interval between IMV and barotraumas, types and course of barotraumas of the included patients.

Each barotrauma event type was recorded as one or multiple barotrauma events occurring on the same day were considered a single event, while separate multiple events were recorded when occurring separated by at least 24 h.

Ventilator settings were not recorded, as outside the scope of this observational study and care was delivered in the same hospital system, with the same resources and management protocol.

### Imaging review

After anonymization, the images for patients with COVID-19 infection suspected barotrauma were reviewed to readers on picture archiving and communications systems (PACS) on 6 megapixels (3280 × 2048 pixels) Barco LCD monitor by three experienced radiologists in consensus (M.A., G.I., and I.A. with 17, 15, and 13 years of experience, respectively) who were blinded to all patients data (Further review will be undertaken by the fourth radiologist in case of disagreement) and to confirm the radiographically reported date and type of barotrauma.

MDCT of the chest were done during the period of our study in CT Room will also evaluated by two experienced radiologist on PACS on Barco monitors.

To exclude that the barotrauma related to line placement or surgical procedure, findings apparent for the first time on a film immediately subsequent to a procedure were excluded.

Patient disposition was classified into three categories as (discharged, still in hospital and died).

We focused on radiological findings and did not investigate ventilator settings.

### Ethics approval and consent to participate

Approval for this study was obtained from the Research Ethics Committee of our medical institute. All study procedures were carried out in accordance with the Declaration of Helsinki regarding research involving human subjects. Written consent was waived.

## Results

Between March 1, 2020 and November 1 2020, 5865 patients older than 20 years seen in our emergency department were complaining for different chest symptoms as cough and chest tightness with or without general symptoms as fever and body ache underwent to X-ray chest and undergoing nasopharyngeal swab testing for SARSCoV-2. Of the 2765 patients who tested positive, 613 were admitted, and 103 were shifted to ICU because of severe pneumonia and acute respiratory failure then progressed to respiratory failure requiring IMV (16.8%). Noted 18 patients,from the 103 patients included in our study, were initially tested negative later became positive for SARSCoV-2.

This was retrospective study for 103 intensive care confirmed COVID-19 infectious patients with respiratory manifestations had barotraumas events admitted to our isolated hospital, their mean age was 56.6, ranged from 21 to 85 years old, 76/103 were males (73.79%) and 27/103 were females (26.21%).

All patients subjected to serial of chest X-ray films and 37/103 patients underwent CT chest (9/37 patients—9.2%) for suspicious of pneumomediastinum, (6/37 patients—16.22%) worsen their condition, (22/37 patients -59.46%) for detection of the extension of barotraumas.

The rate and nature of barotrauma events were presented in Tables 1, 2 and 3.

**Table 1:** Barotraumas event type in the studied patient sample, (NB: one or multiple barotrauma events occurring on the same day were considered a single event, while separate multiple barotrauma events were recorded when occurring separated by at least 24 h)

Barotraumas event type (*n* = 103)			
Single event		Separate multiple events	
Number	%	Number	%
95	92.23	8	7.77

**Table 2:** Types of single barotraumas events

Single event (*n* = 95)					
One barotrauma (*n* = 67)			Multiple barotraumas (*n* = 28)		
Type	*n*	%	Type	*n*	%
Right Pneumothorax	22	32.84	Bilateral pneumothorax	1	3.57
Left Pneumothorax	9	13.43	Bilateral pneumothorax and surgical emphysema	2	7.14
Surgical emphysema	28	41.78	Left pneumothorax and surgical emphysema	4	14.29
Pneumomediastinum	6	8.96	Right pneumothorax-pneumomediastinum	1	3.57
pneumopericardium	2	2.99			
			Bilateral surgical emphysema,right pneumothorax and pneumomediastinum	1	3.57
			Surgical emphysema, left pneumothorax and pneumomediastinum	3	10.71
			Surgical emphysema, bilateral pneumothoraces and pneumomediastinum	4	14.29
			Surgical emphysema, pneumomediastinum and pneumopericardium	1	3.57
			Surgical emphysema pneumoperitononum left pneumothorax and pneumomediastinum	1	3.57
			Surgical emphysema, pneumomediastinum and pneumoperitonium	1	3.57
			Surgical emphysema and Pneumomediastinum	8	28.57
			Surgical emphysema and Pneumoperitonium	1	3.57

**Table 3:** Types of separate multiple events

Separate multiple events (*n* = 8)		
Types	*n*	%
Pneumoperitonium and surgical emphysema then development of large bilateral pneumothoraces 4 days later	1	12.5
Right pneumothorax then left pneumothorax 1 day later	2	25
Right pneumothorax then surgical emphysema 2 days later	1	12.5
Right pneumothorax then pneumomediastinum 2 days later	1	12.5
surgical emphysema then right pneumothorax 1 day later	1	12.5
-Pneumomediastinum and surgical emphysema then left pneumothorax 3 days later	1	12.5
Pneumomediastinum and pneumoperitoneum then surgical emphysema 3 days later	1	12.5

Types of barotrauma events for the studied patients (Table 1) were classified into single event (95/103 patients -92.23%) which one or multiple barotrauma events occurring on the same day and separate multiple barotrauma events (8/103–7.77%) were recorded when occurring separated by at least 24 h.

**Fig. 1:**
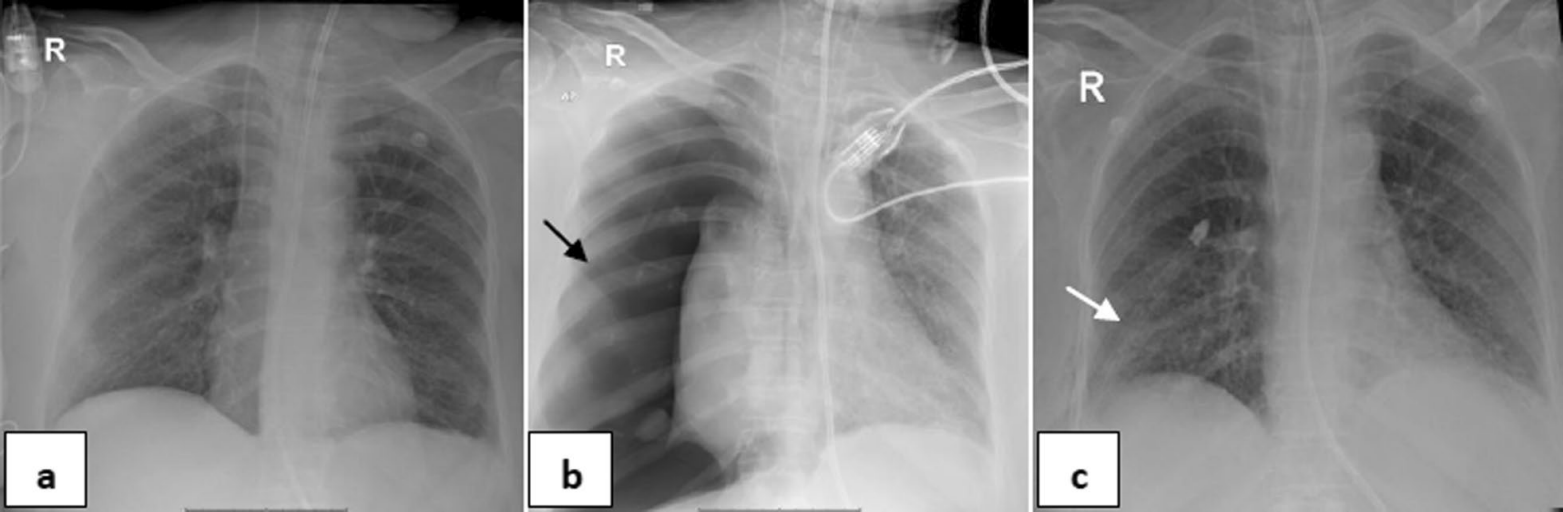
46-year-old male with no previous medical history, intubated 14 days post admission. **a–c** series frontal chest x-ray radiographs demonstrate a large right pneumothorax 4 days after intubation (black arrow). There is a right pleural pigtail catheter (white arrow) and re-expansion of the right lung. Note hazy interstitial densities throughout his lungs

**Fig. 2:**
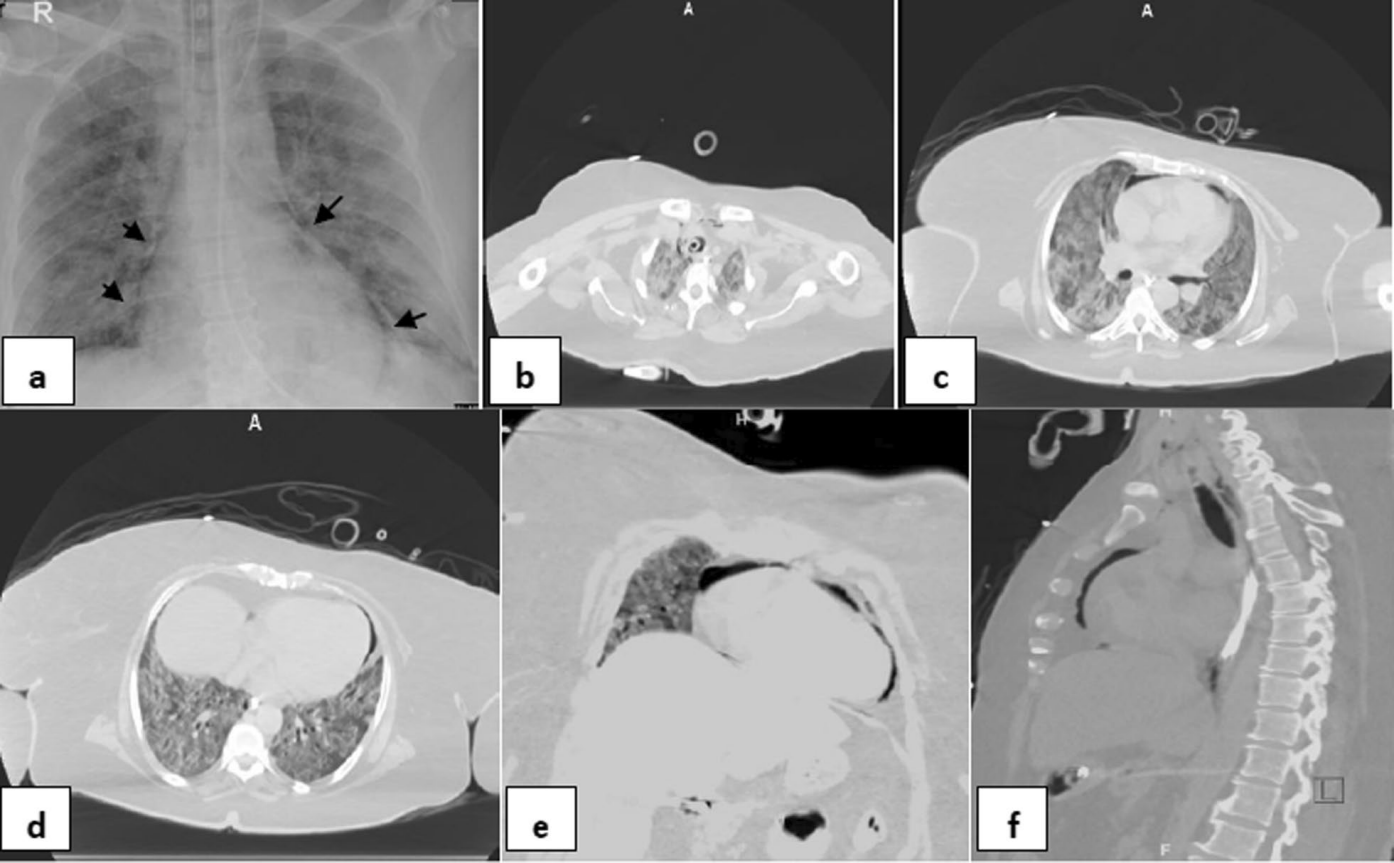
52-year-old female without significant medical history was intubated 7 days after admission, and developed pneumopericardium 4 days after intubation.** a** Frontal chest radiograph depicts mediastinal air bilaterally not exceed to the root of great vessels (black arrows) indicates air within pericardial sac. Note diffuse hazy interstitial lung markings and ill-defined air space opacities bilaterally**. b–d** series axial CT cuts of the chest, **e** and** f** coronal and sagittal reconstructions images, respectively, confirming the X-ray finding

**Fig. 3:**
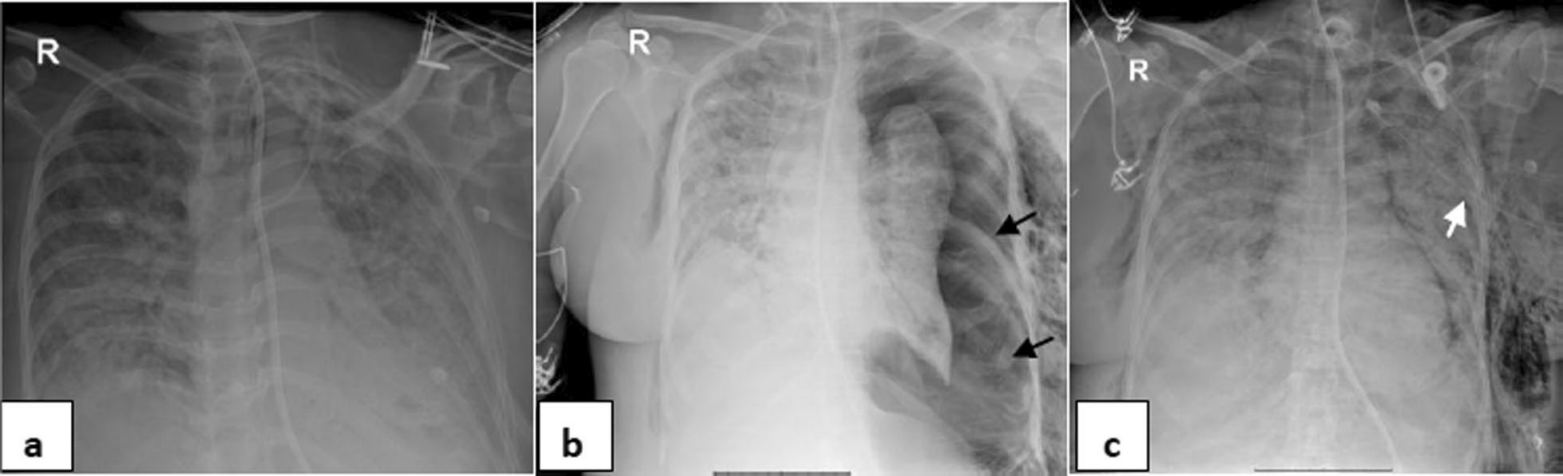
54-year-old female, intubated 12 days post admission. **a–c** series frontal chest X-ray radiographs demonstrates a large left pneumothorax and surgical emphysema 6 days after intubation (black arrows). There is a left pleural pigtail catheter (white arrow) and re-expansion of the left lung. Note diffuse bilateral ill-defined air space densities throughout both lungs

**Fig. 4:**
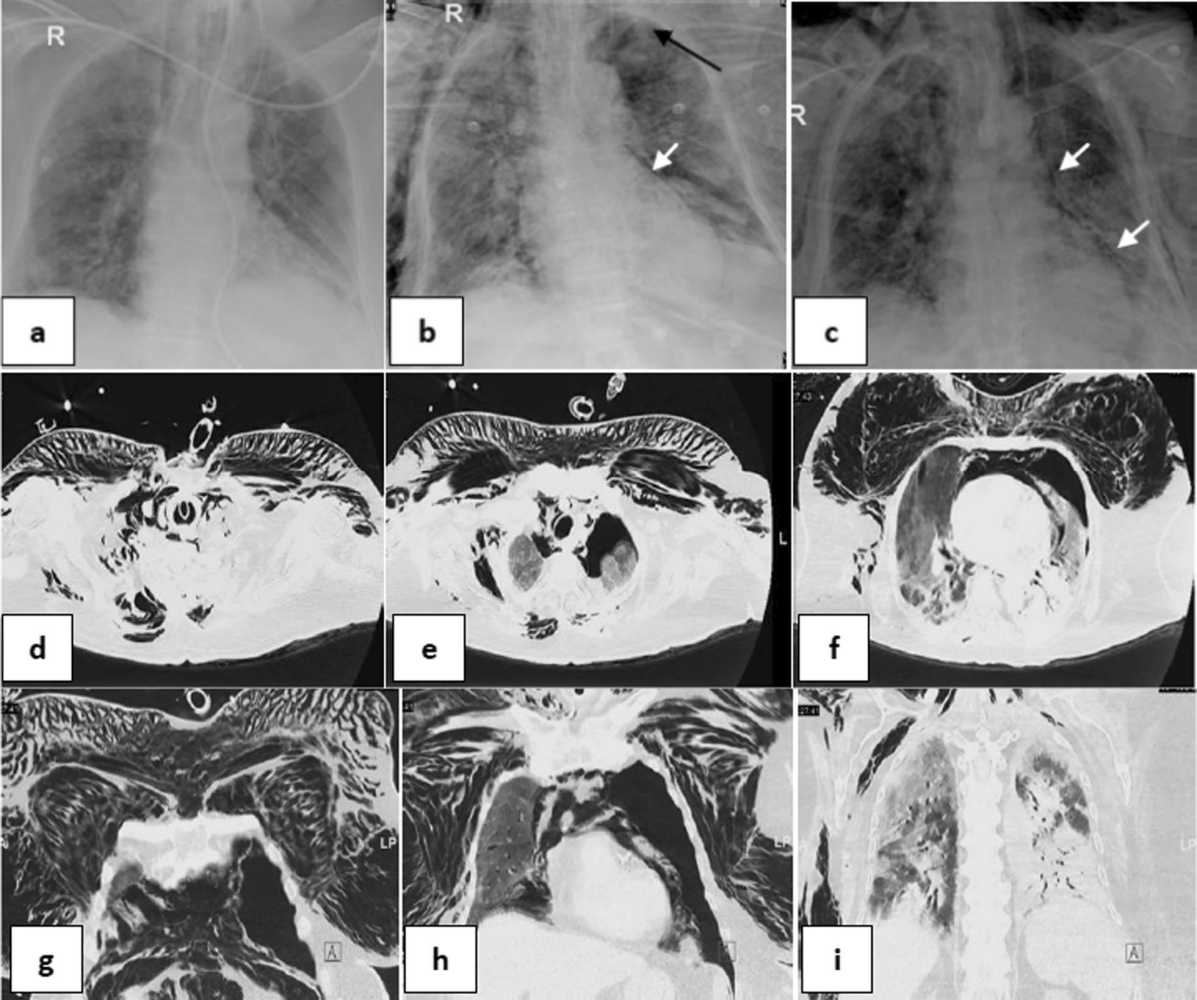
61-year-old male with hypertension and diabetes, intubated 5 days post admission. **a–c** serial frontal chest X-ray films demonstrate a left pneumothorax (black arrow), pneumomediastinum (white arrows) and surgical emphysema 8 days after intubation then progression of surgical emphysema. Note bilateral ill-defined air space opacities throughout both lungs. **d–f** & **g–i** axial and coronal reconstructing CT serial images, respectively, confirming the left pneumothorax, pneumomediastinum and extensive surgical emphysema

**Fig. 5:**
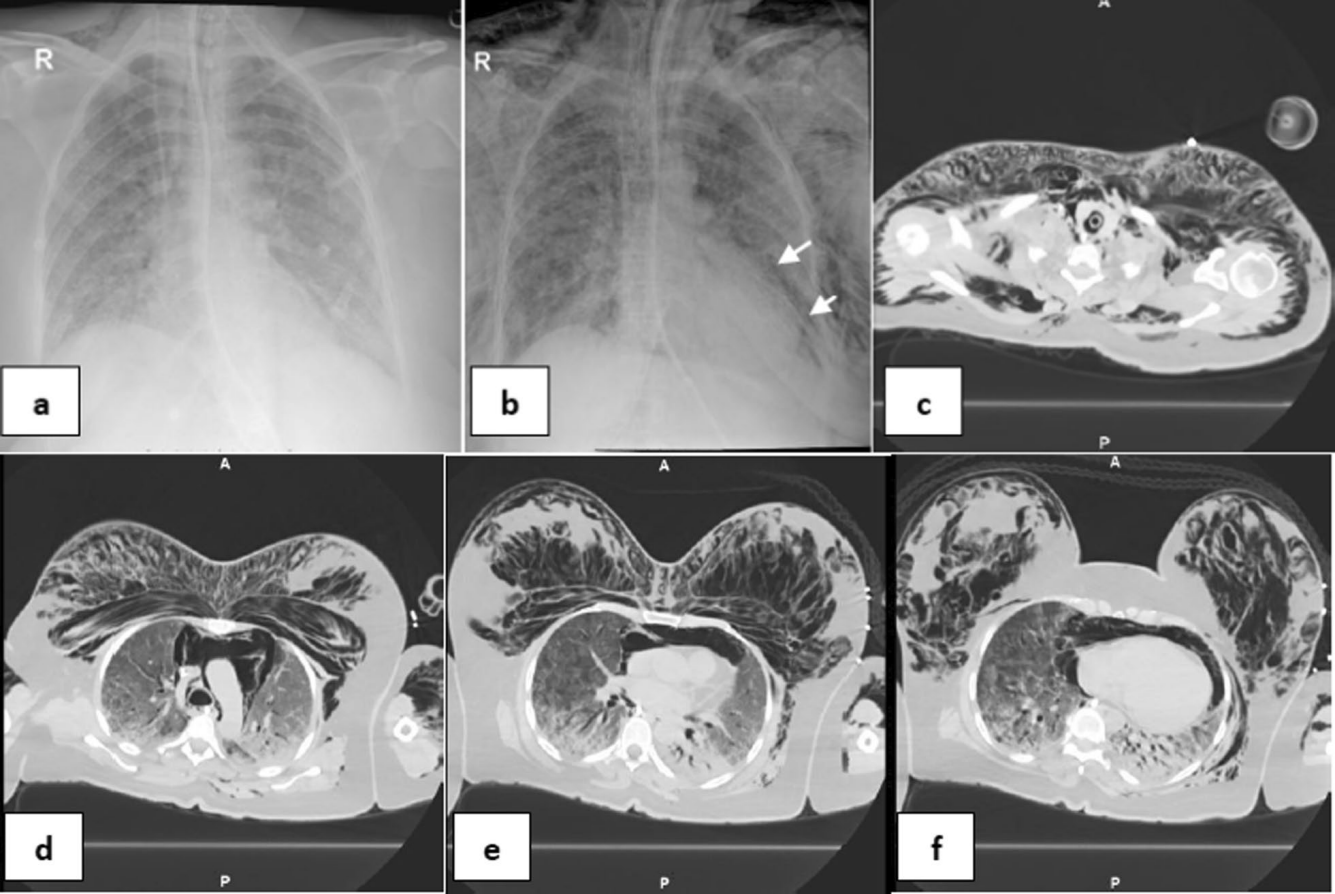
64-year-old female with hypertension and diabetes, intubated 3 days post admission. **a, b** Two serial frontal chest X-ray films demonstrate a pneumomediastinum 4 days after intubation (white arrows), and bilateral chest walls surgical emphysema. Note bilateral ill-defined air space opacities throughout both lungs more in left lower zone.** c-f** series axial CT cuts confirming the pneumomediastinum and bilateral extensive chest wall surgical emphysema

**Fig. 6:**
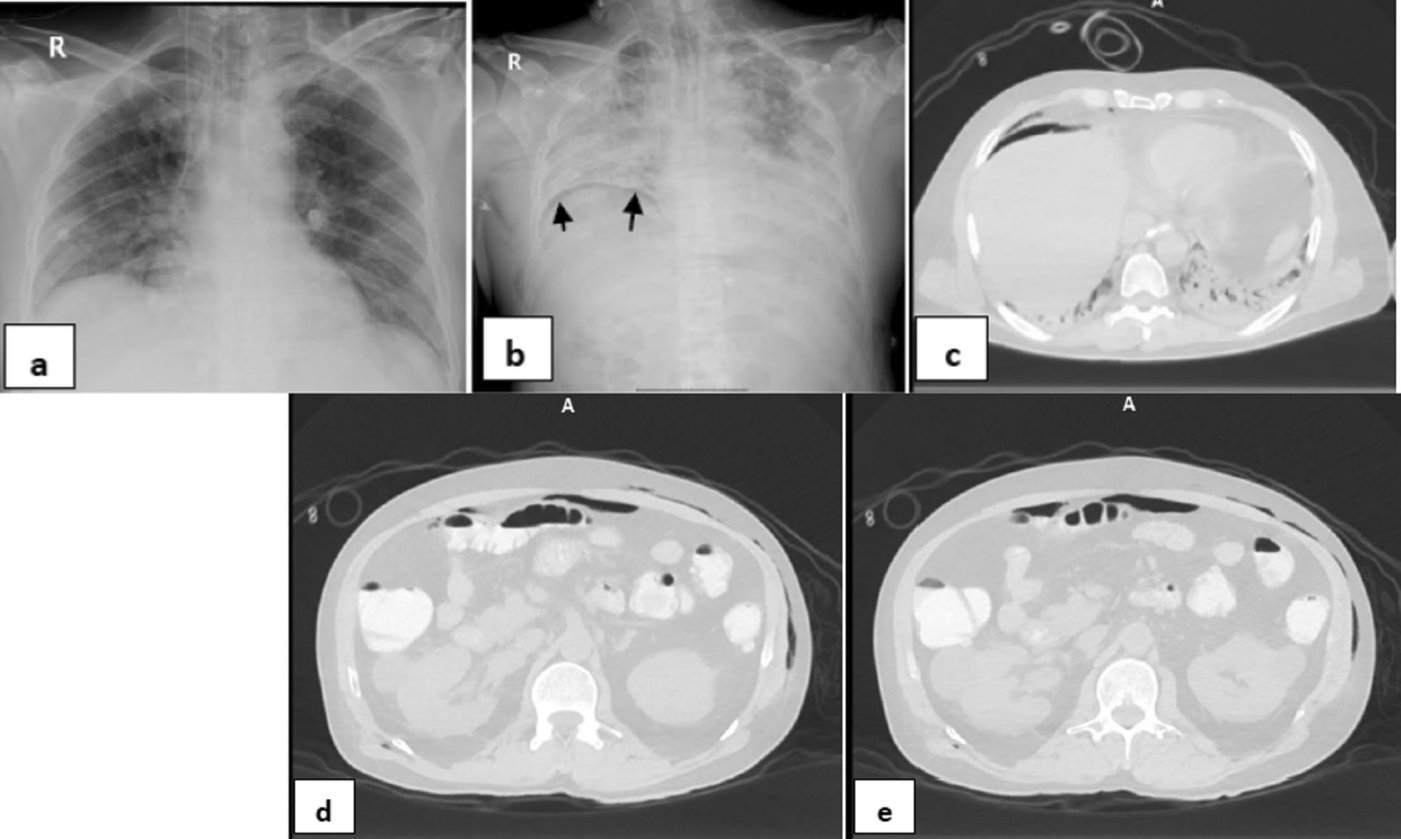
45-year-old male intubated 5 days after admission. **a, b** Two frontal chest radiographs show air under right diaphragmatic copula (black arrows). **c–e** series axial CT abdominal cuts demonstrate peumoperitonium and left lateral anterior abdominal wall surgical emphysema

*Single barotrauma events* were subdivided into (Table 2): one event (67/95 patients—70.53%), included: right pneumothorax (22 patients—32.84%) (Fig. 1), left pneumothorax (9 patients—13.43%), surgical emphysema (28 patients-41.79%), pneumomediastinum (6 patients- 8.96%) and pneumopericardium (2 patients, 2.99%) (Fig. 2) & multiple events (28/95 patients—29.47%), included: Bilateral pneumothorax (1patient—3.57%), bilateral pneumothorax and surgical emphysema (2 patients—7.14%), left pneumothorax and surgical emphysema (4 patients—14.29%) (Fig. 3), right pneumothorax and pneumomediastinum (1 patient—3.57%), bilateral surgical emphysema, right pneumothorax and pneumomediastinum (1 patient—3.57%), surgical emphysema, left pneumothorax and pneumomediastinum (3 patients—10.71%) (Fig. 4), surgical emphysema, bilateral pneumothoraces and pneumomediastinum (4 patients—14.29%), surgical emphysema,pneumomediastinum and pneumopericardium (1 patient—3.57%),Surgical emphysema pneumoperitononum left pneumothorax and pneumomediastinum (1 patient—3.57%), surgical emphysema, pneumomediastinum and pneumoperitonium (1 patient—3.57%), surgical emphysema and pneumomediastinum (8 patients-28.57%) (Fig. 5) and surgical emphysema & pneumoperitonium (1 patient—3.57%) (Fig. 6).

**Fig. 7:**
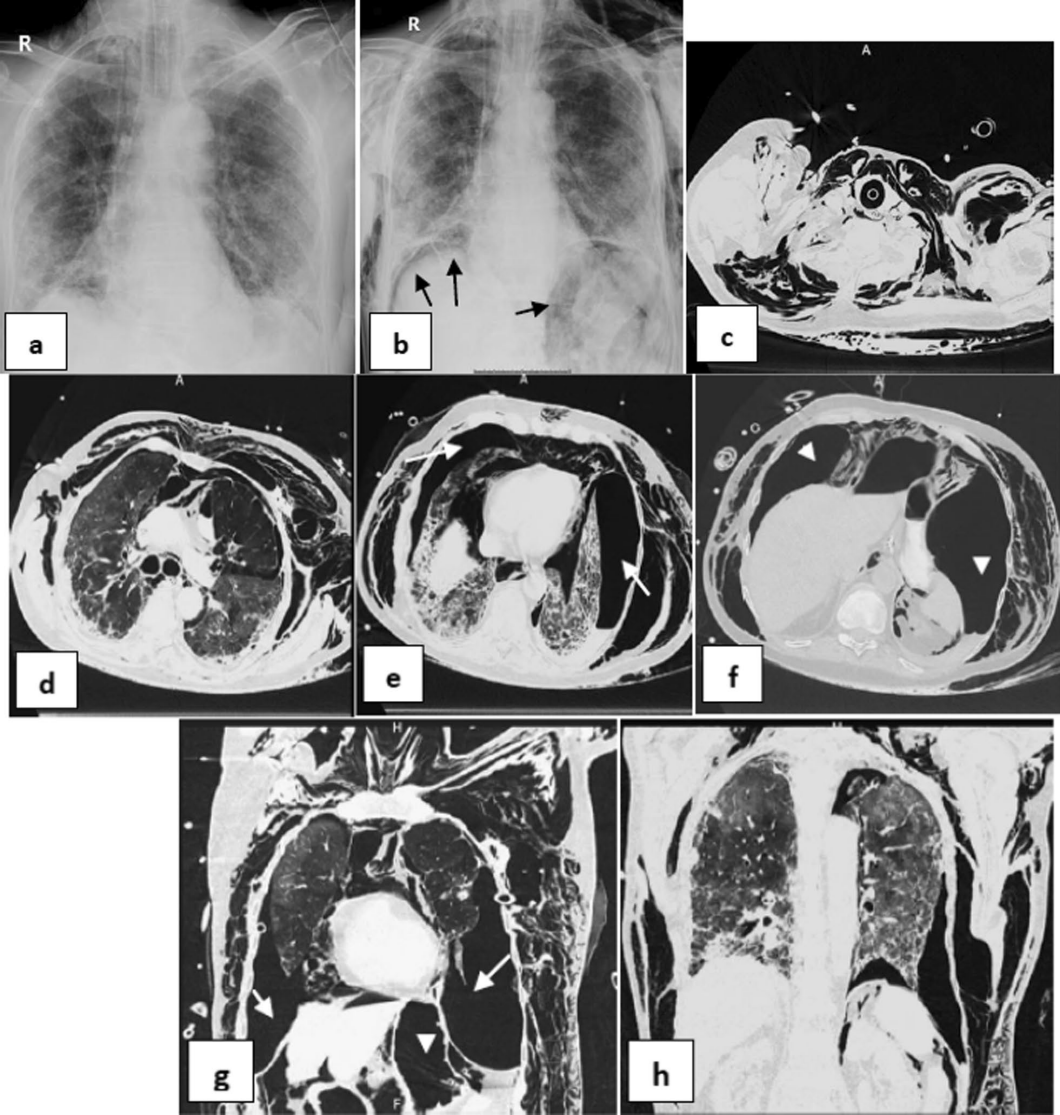
70-year-old male intubated 5 days after admission. **a, b** Two frontal chest radiographs depict moderate pneumperitonium (black arrows) and subcutaneous emphysema. Note bilateral diffuse mainly lower zones bilaterally ill-defined air space opacities with peripheral predominance. **c–f** series axial CT cuts, **g, h** coronal reconstructing images. Four days later, he developed large bilateral pneumothoraces (white arrows), extensive subcutaneous emphysema and moderate pneumoperitonium (white arrow heads)

**Fig. 8:**
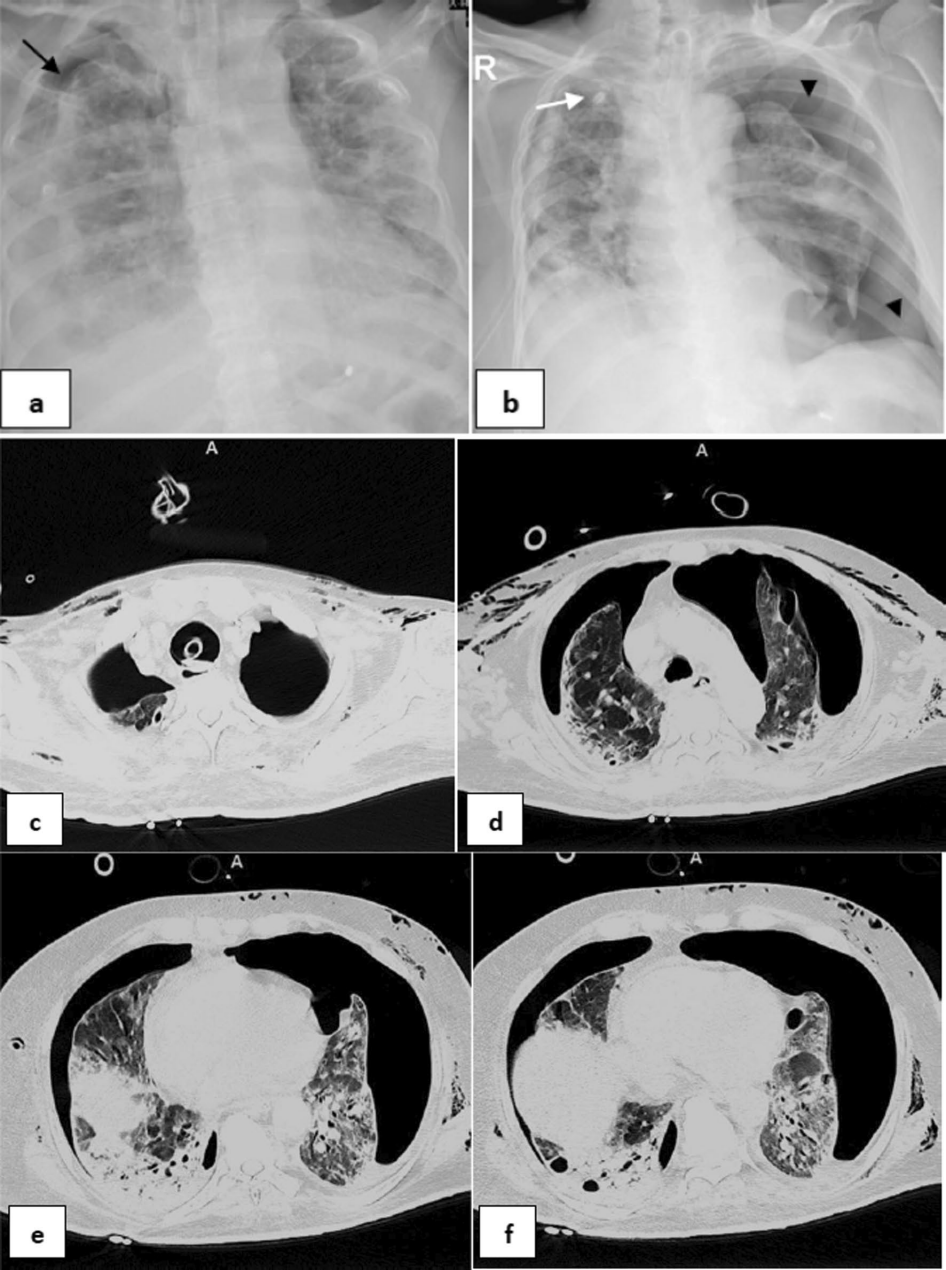
64-year-old female, intubated 5 days post admission. **a**, **b** series frontal chest X-ray radiographs demonstrates a small right pneumothorax 9 days after intubation (black arrow), there is a right pleural pigtail catheter (white arrow) followed by marked left pneumothorax (black arrow heads) 1 day later.Note diffuse bilateral ill-defined air space densities throughout both lungs. **c–f** series axial CT confirming bilateral pneumothoraces

*Separate multiple events* included (Table 3): pneumoperitonium and surgical emphysema then development of large bilateral pneumothoraces 4 days later (1 patient—12.5%) (Fig. 7), right Pneumothorax then left pneumothorax 1 day later (2 patients—25%) (Fig. 8), right pneumothorax then surgical emphysema 2 days later (1 patient—12.5%), right pneumothorax then pneumomediastinum 2 days later (1 patient—12.5%), surgical emphysema then right pneumothorax 1 day later (1 patient—12.5%), pneumomediastinum and surgical emphysema then left pneumothorax 3 days later (1 patient—12.5%) and pneumomediastinum and peumoperitonium then surgical emphysema 3 days later.

The major event was pneumothorax representing 55/103 (53.4%), 31/67 (46%) in one single barotraumas event, {right side 22/67 (32.84%), left side 9/67 (13.43%)}, 17/28 (60.7%) in multiple single barotraumas event and 7/8 (87.5%) in separate multiple events.

Pneumothorax were also classified into non-tension pneumothorax 48/55 (87.3%) and tension pneumothorax 7/55 (12.7%).

The time between invasive mechanical ventilation and the first documented episode of barotrauma was range from 3 to more than 26 days. The majority of the included patients manifested by barotraumas after 3–7 days from intubation 41 patients (39.98%), while 38 patients (36.89%) in 8–14 days, 16 patients (15.53%) in 15–20 days, 4 patients (3.88%) in 21–26 days and 4 patients (3.88%) in more than 26 days.

Patients with barotrauma secondary to COVID-19 respiratory failure were 13 patients (13/103) and they were managed by a single thoracic surgery service.

The clinical characteristics of the included patients were demonstrated in (Table 4), the majority of these patients had underlying comorbidities with HTN and DM being the most common (45/103 patients—43.69%) and Asthma (26/103 patients—25.24%).

**Table 4:** Clinical characteristics of the studied patients

	Number of the patients (*n* = 103)	Percentage
**Smoking history**		
Smoker	36	34.95
X-smoker	9	8.74
Never Smoker	47	45.63
Unknown	11	10.68
**Body mass index (range-Kg/m**^**2**^**)**		
Normal: 18.5–25	44	42.72
Overweight: 25–30	52	50.49
Obese: over 30	7	6.8
**Underlying lung disease**		
Asthma	26	25.24
COPD	7	6.8
Chronic interstitial lung disease	17	16.50
None	53	51.56
**Medical history**		
DM & Hypertension	45	43.69
Cardiac disease & Hypertension	20	19.42
CABG	2	1.94
Chronic Renal Disease	3	2.91
None	33	32.04

Overweight was noted in 52/103 patients (50.49%) which was common and may be a risk factor for respiratory failure leading to invasive mechanical ventilation.

While smoking is a risk factor for barotrauma, the majority of barotrauma patients with COVID-19 infection were never smokers (47/103 patients—45.63%).

The majority of course of barotraumas in the studied patient was regressive after interventional management (13/103 patients—12.6%), while the 11/103 patients with progressive course represented (10.7%) had past medical history (cardiac, hypertension, diabetes mellitus or asthma) and suffering from obesity. Mixed interval changes in multiple barotrauma events were in 24/103 patients—23.30%.

Ninety-six (93%) of included COVID-19 infection patients with barotraumas were associated with prolonged hospital stay were observed in old age (70/103—68%), all patients with separate multiple events (8/103—7.8%), overweight patients (48/103—46.6%), patients with underlying chest diseases (50/103 -48.5%) and patients with other medical problems (70/103—68), the hospitalization period was more than 25 days, while 4 patients died after 5 days from hospitalization and remaining 3 patients had one barotrauma events were regressive course and stay less than 25 days in hospital,high barotrauma rates in patients with coronavirus disease 2019 infection on invasive mechanical ventilation is associated with a longer hospital stay and is a risk factor for higher mortality.

Patient disposition was classified into: 21 patients (20.4%) had been discharged from the hospital, 14 patients (13.6%) were still in hospital and 68 patients (66%) died***.***

Epidemiologic profile and clinical characteristics of died and alive patients are summarized in (Table 5).

**Table 5:** Epidemiologic profile and clinical characteristics of died and alive patients

Demographic and clinical parameters	All patients (*n* = 103)	Died patients (*n* = 68)	Alive patients (*n* = 35)
Sex (Male / Female)	76/27	51/17	25/10
Age (years), mean / range	56.6 /21–85	60.6 / 39–85	48.9 / 21–71
Barotraumas event type:			
Single event	95/103	61/95 (64.2%)	34/95 (35.8%)
One Barotrauma	67/95	42/67 (62.7%)	25/67 (37.3%)
Multiple Barotraumas	28/95	19/28 (67.9%)	9/28 (32.14%)
Separate Multiple Events	8/103	7/8 (87.5%)	1/8 (12.5%)
Interval between IMV and barotraumas 3–7 days	41/103	17/41 (41.5%)	24/41 (58.5%)
Clinical characteristics			
Smoker	36/103	28/36 (77.8%)	8/36 (22.2%)
Obesity	7/103	5/7 (71.43%)	2/7 (28.57%)
Underlying Lung Disease	50/103	39/50 (78%)	11/50 (22%)
Medical history	70/103	53/70 (75.7%)	17/70 (24.3%)

## Discussion

The most common complications in COVID-19 infections are bilateral pneumonia which may progressed to ARDS, complications are more in serious sickness versus non-extreme illness [4].

Positive pressure ventilation is a non-physiological and invasive intervention that can be lifesaving in COVID-19 patients. Similar to any other interventions, it can cause its own danger and complications as it can prompt ventilator-induced lung injury and barotrauma [5].

In this study, we detected a high incidence of barotrauma in patients with coronavirus 2019 infection receiving invasive mechanical ventilation at our isolated hospital during the peak of the COVID-19 pandemic, with a per-total patient admission rate of 16.8%. Udi1 J. et al. [9] reported a rate of barotrauma of 40% in a mixed collective of invasive ventilated COVID-19 patients**.**

Most patients on IMV had at least daily films, but varying intervals between films in this retrospective study could theoretically confound our assessment of single versus separate multiple events.

These high barotrauma rates raise questions of whether coronavirus infections uniquely increase barotrauma risk. At first, it was widely supposed that respiratory failure in COVID-19 infection patients was due to viral pneumonitis progressed to ARDS; thus, many severely ill patients were mechanically ventilated with high positive end-expiratory pressure.

Mechanical ventilation can lead to expansion and increased pressures in the alveoli units leading to inflammatory changes and possibly rupture and spillage of air into the extra-alveolar tissue that can manifest as pneumothorax, pneumomediastinum, pneumoperitoneum, and subcutaneous emphysema [5].

Our observed high rate of barotrauma in COVID-19 patients on IMV may support emerging theories of lung damage in SARS-CoV-2 infection.

Fifty-five patients had either a unilateral or bilateral pneumothoraces, for an overall pneumothorax rate of 55 of 103 (53.4%), McGuinness G. et al., [7], observed overall pneumothorax rate of 54 of 601 (9%). While in reported small study, a pneumothorax rate was 30% in intensive care unit–intubated patients [10].

In the retrospective single-center study observed by Chen et al. [11] including 99 a total of patients with COVID-19 pneumonia, pneumothorax occurred only in 1 patient.

The management of most simple pneumothorax in mechanically ventilated patients involved the placement of a thoracostomy tube to evacuate the air.

In patients with clinically significant pneumothorax, would present with acute vital signs changes, including hypoxia, tachypnea, and tachycardia. Patients may also progress to tension pneumothorax [6]. In patients with tension pneumothorax, management is essential before obtaining a chest radiograph, which requires urgent needle decompression to relieve the pneumothorax, followed by thoracotomy tube placement. In patients presented with a less critical complication, such as a simple pneumothorax with stable vital signs, pneumomediastinum, or subcutaneous emphysema, the clinician should obtain a chest radiograph immediately [6].

Twenty-eight of the 103 patients (27.2%) had pneumomediastinum, while in McGuinness G. et al. [7], represented 10%. As pneumomediastinum is caused by an increase in intra-alveolar pressure, produced alveolar rupture, and air migrates that dissects the peribronchial and perivascular sheaths of the pulmonary hilum (Maclin effect) [12].

Although the smoking represents a risk factor for barotrauma, the majority of intubated barotrauma patients with COVID-19 in our study were never smokers, our result agreed with McGuinness G. et al. [7] observation**.**

In China high smoking rates were initially thought to pool to severe morbidity in early reports of COVID-19 infection; however, in our institution, smoking was not associated with an increased risk of hospitalization or critical illness [13].

In our study, COVID-19 infectious patients with barotraumas were old, suggesting an age-related risk for barotrauma. Our observation was older age in COVID-19 patients after IMV was an independent risk factor for barotrauma. This observation did not agree with Petersen GW [14] study, younger age has been related to barotrauma in patients in the intensive care unit, and barotrauma in McGuinness et al. [7], cohort study was less likely to occur in older patients.

Actually, multinomial regression analysis showed that older patient with barotrauma, is associated with higher mortality.

Our registry detected specific disease conditions; including interstitial lung disease (ILD), chronic obstructive pulmonary disease (COPD), pneumonia, asthma, and acute respiratory distress syndrome (ARDS) represented 50/103 (48.5%) were associated with higher incidence of pulmonary barotrauma. These diseases are concomitant with either poor lung compliance or dynamic hyperinflation, both of which lean patients to elevated alveolar pressure and therefore barotrauma [15].

Patients who develop barotrauma secondary to mechanical ventilation also end up staying in the ICU and on mechanical ventilation for a more broadened period. Longer time on mechanical ventilation may result in additional complications secondary to barotrauma as well as others, including ventilator-associated pneumonia, delirium, intensive care acquired weakness, and nosocomial infections [16].

Whereas, our survival rate from patients with barotrauma was 20.5%, indicating that in most cases barotrauma were severe, complicated and could not successfully be managed and was an inevitable cause of death in our cohort of patients, while Udi et al. [9] study showed survival rate was 75%.

At the end of our period, 13.6% COVID-19 patients with barotrauma remained hospitalized, while represented 30% in McGuinness et al. [7], cohort study and it was not clear whether barotrauma caused prolonged hospitalization or longer hospital stay was a risk for barotrauma.

Breathing natural mechanism in humans depends upon negative intra-thoracic pressures. In contrast, mechanically ventilate patients are on with positive pressures. Since positive pressure ventilation is not physiological, it may raise the occurrence of barotrauma [17].

There were few limitations in this study: a small number of the patient population, the study was single-center retrospective study, pathologic correlation was not available for many included patients.

We focused on chest X-ray and CT chest findings and did not investigate a possible association between barotrauma and respirator settings. Since optimum characterization and detection of barotraumas frequently require computed tomography, the findings might be under diagnosed if only less sensitive chest radiography is obtained.

## Conclusion

In conclusion, our study confirmed that barotraumas in COVID-19 patients secondary to invasive mechanical ventilation may reflect a worse prognosis, carried high morbidity and mortality and more closely related to the patient’s underlying medical condition and elder patients.

## Data Availability

The data sets used and/or analyzed during the current study are available from the corresponding author on reasonable request.

## Abbreviations

COVID-19: Coronavirus disease 19
SARA-COV2: Severe acute respiratory syndrome coronavirus
WHO: World Health Organization
ICU: Intensive care unit
CXRs: Chest X-Rays
MDCT: Multi-Detector Computed Tomography
ARDS: Acute Respiratory Distress Syndrome
COPD: Chronic obstructive pulmonary disease
ILD: Interstitial lung disease
IMV: Invasive Mechanical Ventilation
HTN: Hypertension
DM: Diabitis mellitus
